# Changes of the Public Attitudes of China to Domestic COVID-19 Vaccination After the Vaccines Were Approved: A Semantic Network and Sentiment Analysis Based on Sina Weibo Texts

**DOI:** 10.3389/fpubh.2021.723015

**Published:** 2021-11-11

**Authors:** Hao Gao, Difan Guo, Jing Wu, Qingting Zhao, Lina Li

**Affiliations:** ^1^School of Journalism and Communication, Nanjing Normal University, Nanjing, China; ^2^Faculty of Social Sciences, University of Ljubljana, Ljubljana, Slovenia; ^3^Film-Television and Communication College, Shanghai Normal University, Shanghai, China

**Keywords:** COVID-19, vaccines, vaccination, official announcement, public attitude, China

## Abstract

**Introduction:** On December 31, 2020, the Chinese government announced that the domestic coronavirus disease-2019 (COVID-19) vaccines have obtained approval for conditional marketing and are free for vaccination. This release drove the attention of the public and intense debates on social media, which reflected public attitudes to the domestic vaccine. This study examines whether the public concerns and public attitudes to domestic COVID-19 vaccines changed after the official announcement.

**Methods:** This article used big data analytics in the research, including semantic network and sentiment analysis. The purpose of the semantic network is to obtain the public concerns about domestic vaccines. Sentiment analysis reflects the sentiments of the public to the domestic vaccines and the emotional changes by comparing the specific sentiments shown on the posts before and after the official announcement.

**Results:** There exists a correlation between the public concerns about domestic vaccines before and after the official announcement. According to the semantic network analysis, the public concerns about vaccines have changed after the official announcement. The public focused on the performance issues of the vaccines before the official approval, but they cared more about the practical issues of vaccination after that. The sentiment analysis showed that both positive and negative emotions increased among the public after the official announcement. “Good” was the most increased positive emotion and indicated great public appreciation for the production capacity and free vaccination. “Fear” was the significantly increased negative emotion and reflected the public concern about the safety of the vaccines.

**Conclusion:** The official announcement of the approval for marketing improved the Chinese public acceptance of the domestic COVID-19 vaccines. In addition, safety and effectiveness are vital factors influencing public vaccine hesitancy.

## Introduction

The WHO declared the global coronavirus disease-2019 (COVID-19) pandemic on March 11, 2020 (1). There have been ~110.7 million confirmed cases of COVID-19 and over 2.4 million COVID-19 related deaths as of February 23, 2021 (2). Protective actions against the virus, such as wearing masks and keeping physical distance, have radically influenced social life, and even national and household economies (3). Vaccination has been considered the ideal measure of intervention against infectious diseases in the past century. It has also been institutionalized for epidemiological prevention and management in the past 20 years (4). The most promising solution for controlling the pandemic is developing and deploying safe and effective vaccines (5), contributing to herd immunity, and eventually, bringing back the normalization of social life and economic development (3). On this occasion, governments worldwide looked forward to developing and producing vaccines to prevail over the epidemic (6).

China has begun to develop the COVID-19 vaccine at a very early stage after the epidemic outbreak, and the first batch of domestic COVID-19 vaccines obtained clinical trial approval in March. On December 31, 2020, the state council information office of People's Republic of China (PRC) released that the Sinopharm inactivated vaccine from China was approved by the National Medical Products Administration for conditional marketing (7) and was made available for the public to be vaccinated in batches on a voluntary basis. However, from the perspective of pandemic control, herd immunity against COVID-19 needs a substantial proportion of the population to take the vaccines (8). Many factors would affect the result of the vaccination programs promoted by authorized organizations like WHO and governments. When the vaccines are available, the final success of the vaccination program depends on the acceptance of the public of the vaccines (9). The public would easily refuse vaccination due to the unknown and continuous variations of COVID-19 (10). Research related to the flu vaccines indicated that most people were unwilling to accept a new and unapproved vaccine (11). Studies taken during the vaccine development about the willingness of the public on COVID-19 vaccination showed different attitudes among different countries, and the people who were willing to take the vaccines ranged between 41 and 89% (12). So, it is necessary to examine the attitudes of the public toward the approved COVID-19 vaccines.

In the digital age, big data offers new perspectives and approaches to research in many fields, including public health (13). In public health emergencies, online data is used to track the process of epidemics, public concerns, and monitor crises, predict epidemic tendencies, and provide warnings (14). Specifically, in the field of vaccination, scholars have applied big data as an alternative to replace traditional research when big data is in its infancy. Their findings indicated that big data could provide insights into public concerns with more detailed analysis and research, essential to promoting vaccination (15, 16). In terms of the data sources, previous research pointed that real-time monitoring and analyses of vaccination *via* online media reports, such as online blogs, articles, and government reports, are helpful for immunization programs to deal with specific public concerns (17). Furthermore, Twitter provides research data with over 400 million tweets every day, and social media has become an important source of big data (18). A study on Twitter indicated that tweets on Twitter could measure public interests or concerns about health issues (19).

Chinese media has provided timely and follow-up news releases on the development of the COVID-19 vaccines from China at a very early stage. Vaccine-related news has generated great public attention and raised hot discussions on social media platforms like Sina Weibo, where many people leave comments and express attitudes. The official announcement of the COVID-19 vaccine was a matter of self-interest to the public, driving emotional responses and changes among the public in a short term after the announcement. On one hand, social media provides a platform for the Chinese government to track public concerns and demands during the COVID-19 pandemic. On the other hand, social media has allowed the dissemination of prevention information and has influenced public attitudes and behaviors (20). This article aims to examine the public attitudes to the approved vaccines and investigate whether there are changes in their attitudes after the official announcement of the approved COVID-19 vaccines.

The use of social media data to measure groups represents a fundamental shift in measurement methods. Measuring population health behaviors traditionally requires significant resources in time and space. However, online social media provide unprecedented access to data and effective and inexpensive analysis tools for research (21). In terms of attitude study, an attitude refers to an individual evaluation tendency to a target object composed of cognition (opinion, belief, knowledge, expectation, etc.) and behavior tendencies (22). Classification techniques were used to analyze vaccine sentiments *via* social networks in the studies on attitudes toward vaccines from social media contents (15, 23, 24). In addition, the Vaccine Attitude Surveillance using Semantic Analysis (VASSA) framework combines semantic web and natural language processing (NLP) techniques with online data for evaluating and analyzing vaccination attitudes and beliefs (23). Some studies have analyzed vaccine contents from Facebook, Twitter, and YouTube with semantic network analysis (16, 25–28). These studies suggested that linguistic analysis can identify the attitudes or cognitive components of vaccine contents on social media. Previous research has used Twitter data to explore the changes in the public sentiments on pandemic influenza A (H1N1) regarding the sentiments contained in attitudes. The study found that the predicted vaccination rates based on sentiments expressed on Twitter closely matched with those estimated by the Centers for Disease Control and Prevention (CDC) through traditional telephone surveys (21). A study from the USA used text mining and sentiment analysis to research the dimensions of misinformation about the human papillomavirus (HPV) vaccine on Instagram (29). Overall, a growing number of studies have applied big data analysis in studying vaccine content on social media. These studies provided important insights into social media influencing health attitudes and behaviors and inspired the research on Sina Weibo content with big data.

This study took the posts on Sina Weibo as research objectives to explore the Chinese public attitudes to the domestic COVID-19 vaccines. As one of the most influential social media outlets in China, Sina Weibo has 511 million monthly active users and is more open than other Chinese social media (30), which has become the most extensive online public opinion space. Therefore, exploring the attitudes shown on Weibo texts can reflect how the Chinese public viewed the approved COVID-19 vaccines, to a certain extent.

**Figure 1:**
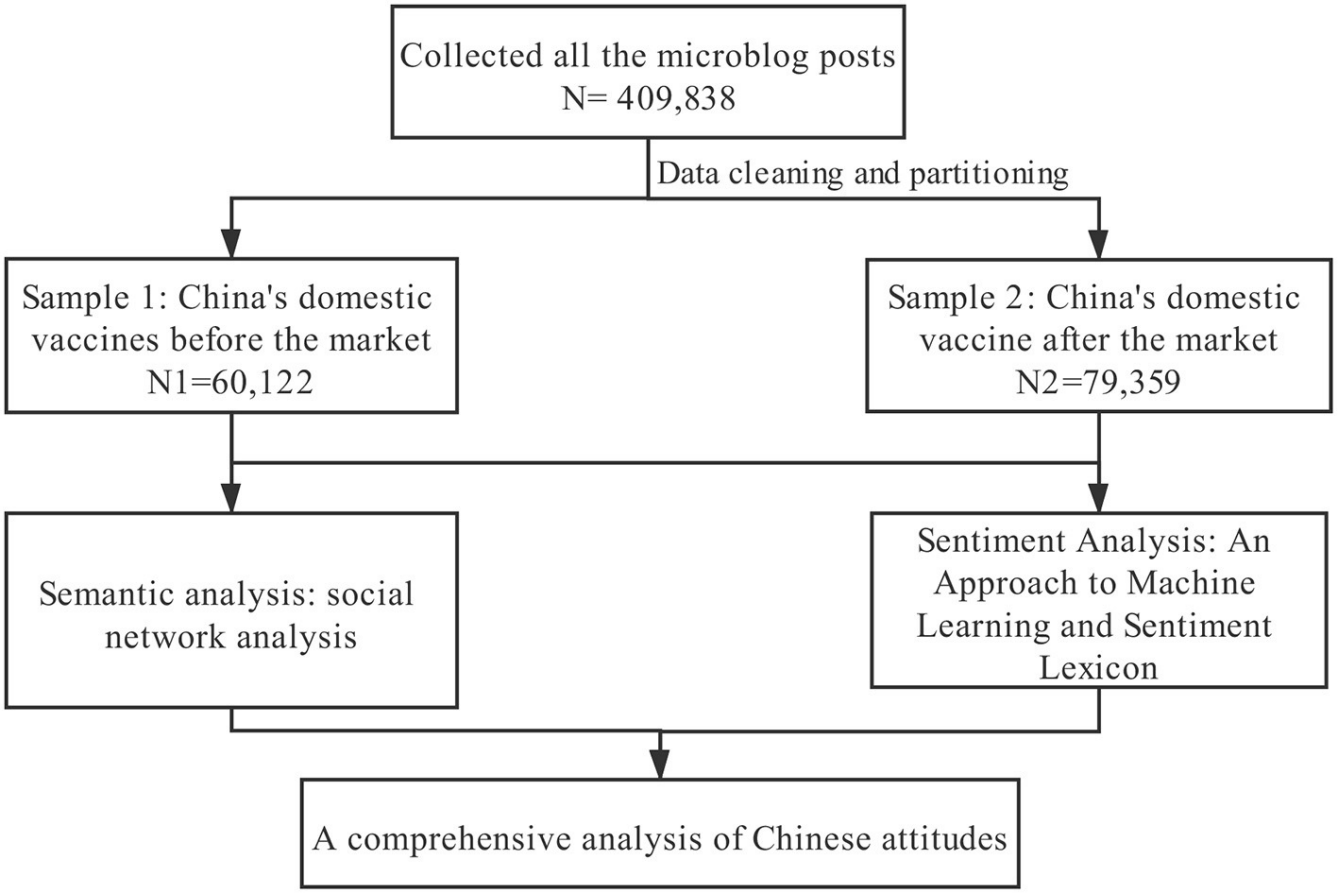
Design of this study.

This article employed semantic network and sentiment analysis based on Sina Weibo texts related to the COVID-19 vaccines *via* data mining. Semantic network analysis analyzed the texts posted by the public and then focuses on public concern about the COVID-19 vaccines. Sentiment analysis was used to judge the emotions reflected in these texts and learn the characteristics of the emotions of the public to the COVID-19 vaccines. Specifically, the Chinese public attitudes to vaccines can be examined by combing the two analysis methods (Figure 1). To check the changes in attitudes, this research selected two groups for comparison, which are the vaccine-related texts posted a week before the official declaration of the approved vaccine (from December 25–31, 2020) and those posted a week after the official statement of the approved vaccine (from January 1–7, 2021).

## Methods

### Data Collection and Cleaning

Discussions about COVID-19 vaccines among the Chinese people remained high since the outbreak of the epidemic in China. They were sparked further after the announcement of the government that the domestic vaccines have been approved for marketing on December 31, 2020. Before collecting data *via* self-written python scripts, we checked the number of posts and text contents under different keyword searches. Compared with other keywords, we found that texts with the keyword “COVID-19 vaccines” were more comprehensive and matching with COVID-19 vaccine-related contents. Thus, this paper determined the research sample as the texts under “COVID-19 vaccines”. As shown in Table 1, this article collected 409,838 Weibo posts with the key search word “COVID-19 vaccines” *via* the Sina Weibo application programming interface (API) from December 25, 2020 to January 7, 2021. With data cleaning, the texts that are repeated, meaningless, and contain pure emojis have been deleted. We defined December 31, 2020 as the critical date to separate the collected data into two samples: sample 1 contains the texts posted 1 week before the crucial date amounting to 60,122, and sample 2 contains those posted 1 week after the critical date amounting to a total of 79,359. Then, we subjected the two samples to semantic analysis and sentiment analysis and investigated the change of the attitudes of Chinese netizens toward domestic COVID-19 vaccines.

**Table 1:** The degree centrality of keywords in two semantic networks.

	**Sample 1**		**Sample 2**	
**NO**.	**Key words**	**Centrality**	**Key words**	**Centrality**
1	Vaccines	44,683	Vaccines	49,221
2	COVID-19	43,694	COVID-19	48,507
3	Vaccination	16,846	Vaccination	40,141
4	China	8,729	Production capacity	19,896
5	Effectiveness	4,398	Large-scale	9,121
6	Safe	4,184	Free	8,159
7	Safety	4,157	Break	5,083
8	Effective	3,823	China	4,572
9	Appreciate	3,812	America	4,521
10	Free	3,616	Emphasis	4,430
11	Highly	3,608	Crowd	4,213
12	Staff	2,908	Nationwide	4,156
13	Crowd	2,815	Satisfy	3,850
14	Virus	2,781	Virus	3,685
15	Precedence	2,733	Deliberately	3,502

### The Semantic Analysis

The semantic analysis in this study used social network analysis based on keyword co-occurrence. The core of this method is to use keywords to summarize the theme of the sample and construct a keyword co-occurrence matrix by counting the frequency of keywords co-occurring within the sample. On this basis, the method can form a keyword co-occurrence network *via* cluster analysis, which is the semantic network (31). This method provides insight into the main content and salient themes of the samples, widely applied in text analysis and information retrieval. Several software packages were available for social network analysis, including UCINET, ROST, and NetDraw. This study first employed ROST, which Prof. Shen developed in Wuhan University in 2010, used by researchers worldwide (32). As a common Chinese social network analysis tool, it can perform high precision semantic network analyses, generate keyword matrices, and draw semantic networks on various text contents such as Weibo, essays, and chat logs. This study also employed UCINET, a Chinese and English social network analysis tool, to calculate the degree centrality and Quadratic Assignment Procedure (QAP).

This study operated the semantic analysis according to the following:

Step 1: Constructing the keyword co-occurrence matrices of samples 1 and 2, respectively with ROST 6.0 (ROST Virtual Learning Team of Wuhan University);
Step 2: Drawing the keyword co-occurrence network graphs of samples 1 and 2 respectively with ROST 6.0;
Step 3: Calculating the degree centrality of the keyword co-occurrence matrices conducted above with UCINET 6.0 (FLin Freeman, Martin Everett and Steve Borgatti);
Step 4: Calculating the correlations (QAP value) between samples 1 and 2 with UCINET 6.0.

### Sentiment Analysis

As the samples in this study were mostly short texts and large in volume, the sentiment analysis was conducted in two stages.

In the first stage, we conducted a rough classification of the sample emotions using the Convolutional Neural Network (CNN). The CNN is a deep learning model that uses the emotional features in the text to identify emotional tendencies. Research has confirmed that CNN has an accuracy of over 90% in the sentiment classification of Weibo texts (33). Convolutional Neural Network classifies sample emotions by constructing a standard sentiment classification database and helping word vectors learn the syntax and semantics of the database. The rough classifications of emotions include dichotomous (positive and negative), trichotomous (positive, neutral, negative), and quadratic (positive, neutral, negative, other) (21). After the data cleaning in this study, there was no irrelevant content, and positive, neutral, and negative contents all have a certain volume. This study divided the samples into positive emotions (including welcome, support, acceptable, joy, and praise for COVID-19 vaccines), neutral emotions (including wait-and-see, hesitation, surprise, and uncertainty about COVID-19 vaccines), and negative emotions (including worry, fear, disgust, suspicion, and anger about COVID-19 vaccines).

The first stage was operated as the following:

Step 1: Using Chinese Orientation Analysis and Evaluation in 2014 (COAE 2014, a corpus contains 7,000 Weibo texts labeled with sentiments, which is commonly used as a training and test set for Chinese sentiment classification) as the subject corpus and manually tagging a little sample as the auxiliary corpus, the two constitute the database (34);
Step 2: Using word2vec for unsupervised word vector learning. Word vectors can obtain syntactic and semantic information during the training process;
Step 3: Testing the training results of the model and ensuring the sentiment classification accuracy was over 80%;
Step 4: Importing samples 1 and 2, respectively, and outputting the three emotional orientations classification results.

In the second stage, we conducted a detailed classification of the sample emotions based on a bag-of-words model, reducing the dimensions of the texts to words. The bag-of-words model is commonly used for sentiment recognition in social media texts such as Weibo and Twitter and has the advantages of high speed and accuracy when processing a large number of short texts (33). This model labeled texts with sentiments by invoking a sentiment dictionary and comparing the dimension-reduced samples with the words in the dictionary one by one. This study employed Emotion ontology of Dalian University of Technology (DLUT-Emotion ontology) as the sentiment dictionary, a mature application in Chinese sentiment classification with over 85% of accuracy (35). The DLUT-Emotion ontology contains nearly 30,000 Chinese sentiment words and divides it into seven layers of Chinese sentiments: Anger, Disgust, Fear, Sadness, Surprise, Good, and Happy. Referring to the classification of the DLUT-Emotion ontology, we finally defined seven specific emotions from the samples with words as the basic unit. Here are the examples of words with different sentiments, Anger (sample words: blame, dare, fury), Disgust (sample words: rigid, stoop, vanity), Fear (sample words: helpless, cramped, disease), Sadness (sample words: miss, lose, cry), Surprise (sample words: rare, shocked, faint), Good (sample words: trust, agree, admire), and Happy (sample words: joy, fun, relaxed). The second stage examined the sample sentiments from the word frequency, complementing the first stage, examining the word order and semantics.

The second stage was conducted according to the following:

Step 1: Reducing the dimensions of samples 1 and 2 to word-level *via* python-jieba;
Step 2: Employing DLUT-Emotion ontology for sentiments classification, testing the accuracy of the bag-of-words model, and ensuring the accuracy is over 80%;
Step 3: Importing samples 1 and 2 and outputting the results of the seven specific emotions classification;
Step 4: A comparison study of the sentiment analysis results of the two stages.

## Results

### Semantic Analysis

**Figure 2:**
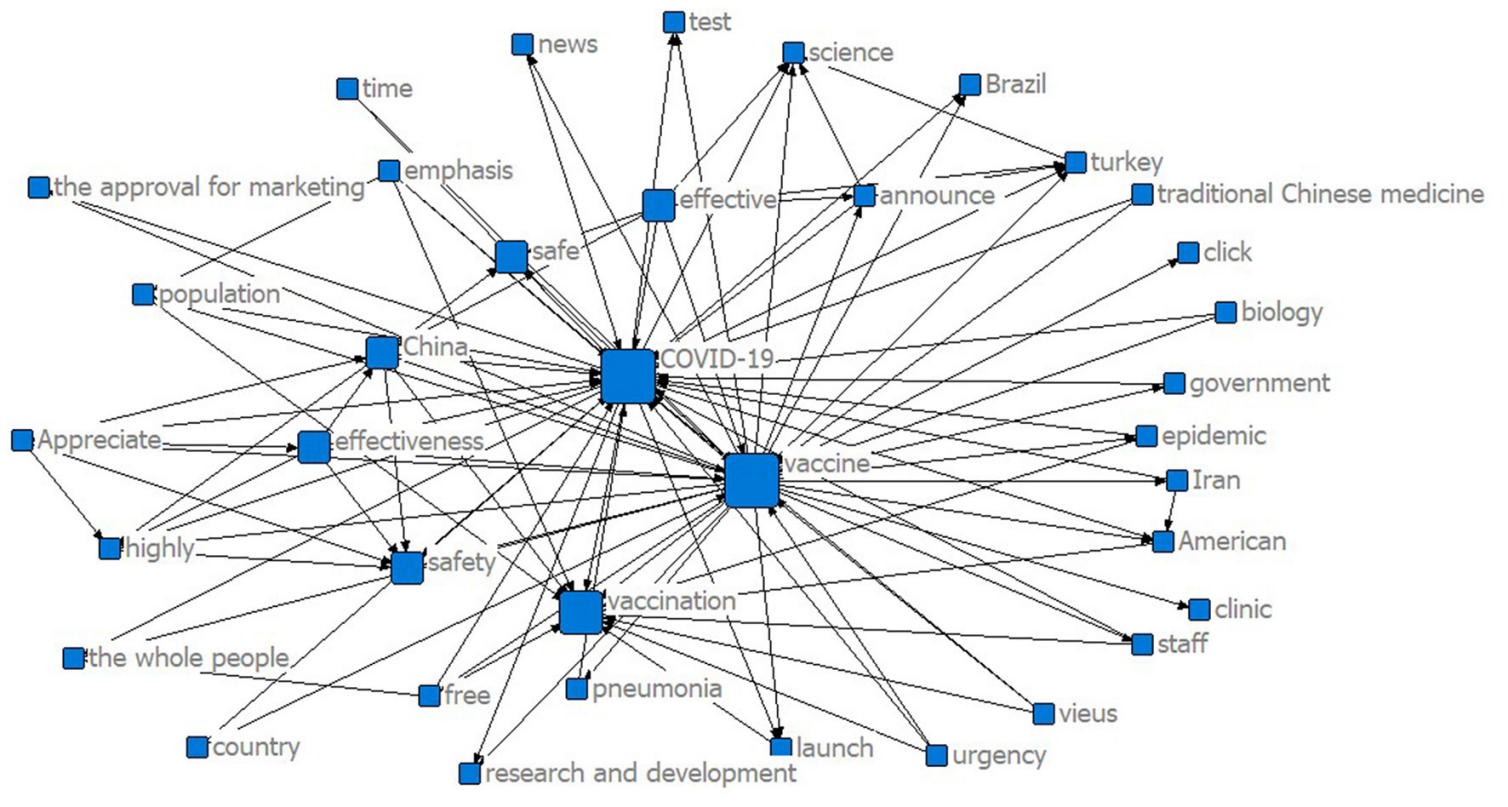
Semantic network graph of domestic COVID-19 vaccines before approved for marketing.

**Figure 3:**
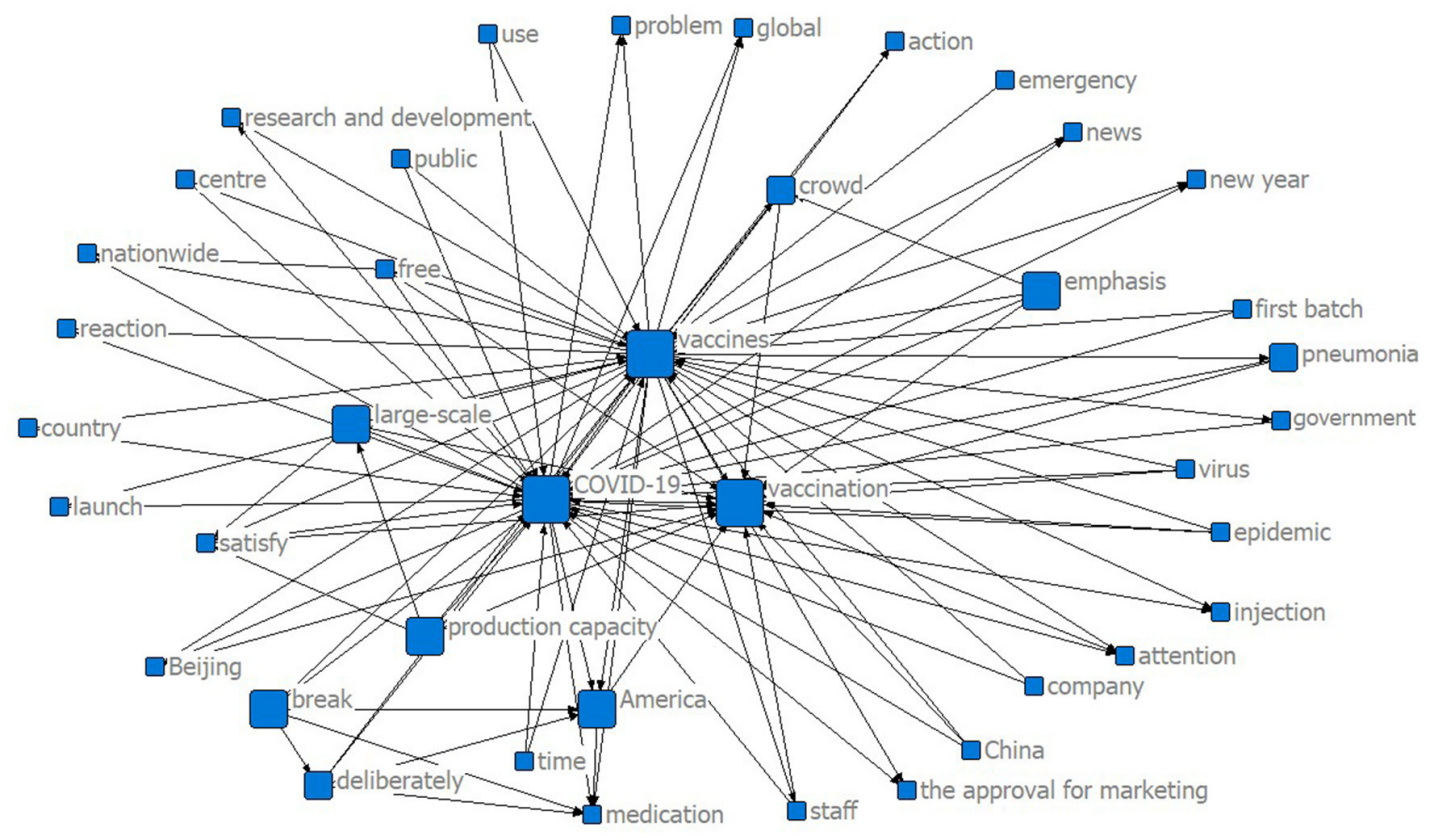
Semantic network graph of domestic COVID-19 vaccines after approved for marketing.

Figures 2, 3 show the two semantic networks according to the semantic analysis. In Figure 2, the keyword co-occurrence network for the domestic COVID-19 vaccines of China before approval for marketing shows that “COVID-19” and “vaccine” have distinctly higher frequencies than other words, and the word “vaccination” ranks in a secondary central position after them. The size of the vertices shows that the words “COVID-19” and “vaccine” also have higher attributes and impact than “vaccination.” Other keywords like “safe,” “safety,” “effectiveness,” “China,” “epidemic,” “population,” and “staff” are positioned in the second-word circle in this graph. This graph shows that before the domestic COVID-19 vaccines were approved for marketing, the Chinese people cared most about the safety of vaccines and paid attention to the safety of vaccination for different groups of people and the effectiveness of vaccines for epidemic control.

Figure 3 shows the keyword co-occurrence network of the domestic COVID-19 vaccines of China after the marketing approval. It is apparent that the three words “COVID-19,” “vaccines,” and “vaccination” have the highest frequency, and the size of the three vertices are similar, indicating that they have similar word centrality too. Words like “production capacity,” “large-scale,” and “nationwide” also have relatively high frequency due to the process of vaccine marketing and promotion, which shows the public concern about the mass production and nationwide dissemination of COVID-19 vaccines. In addition, there have been numerous discourses about comparing domestic vaccines to foreign vaccines, especially those made in the USA and Europe.

The two keyword co-occurrence graphs show what the Chinese public was concerned about most before and after the COVID-19 vaccines were approved for marketing. The public was concerned more about the performance issues of the vaccines when the vaccines were not approved yet, such as the safety, side effects, and effectiveness of the vaccines. After the marketing approval, the public switched their concerns toward the practical issues of the vaccines, such as the vaccination program and vaccine production. They also compared domestic vaccines with foreign vaccines to express their opinions.

We also conducted a semantic network degree centrality analysis in this study. Table 1 shows the degree centrality analysis on the keyword matrices based on the two samples and generates the core words of the two semantic networks. Overall, sample 2 had a higher degree of centrality in core words than sample 1, which indicates that with the progress of the approved vaccines for marketing, the public concern is more concentrated, and the core keywords are more prominent. The performance and practical issues of the vaccines gained public attention both before and after the marketing approval.

Issues on the safety and effectiveness of the vaccines and the priority vaccination groups gained more public attention before the marketing approval. According to the Weibo contents, the public questioned the safety of the domestic COVID-19 vaccines as they have not entered phase III clinical trial at that time. The effectiveness of the vaccines has not been tested and proved, which caused public hesitancy and resistance to the vaccines. Although there existed public concerns about the safety and effectiveness of the vaccines, which groups of people should be given the priority for vaccination has raised hotter discussion among the public. Many believed that people who work in hospitals, railway stations, communities, and other frontline positions should be prioritized for vaccination.

After the vaccines were approved for marketing, practical issues of the vaccines, such as the vaccination program, the capacity, and the scale of vaccine production, drove the public attention. The centrality of the word “vaccination” reached 40,141, which is more than twice before the marketing approval. The netizens discussed the advantages of the domestic COVID-19 vaccines, the process of the vaccination appointment, the precautions of vaccination, and the potential side effects caused by the vaccines. There are another two trending topics after the vaccines were made available. On one hand, the Chinese government announced a policy on free vaccination of domestic COVID-19 vaccines for all Chinese citizens, which triggered intense debates and increased the vaccination willingness to some extent with public praise for the policy. On the other hand, netizens compared domestic vaccines with foreign-produced vaccines such as Pfizer, Modena, and AstraZeneca in terms of the development technology, safety, effectiveness, side effects, and existing cases of adverse reactions to vaccination.

Finally, we explored the relations between the two semantic networks. As shown in Table 2, the QAP analysis showed a significant positive correlation between the two semantic networks in this study, with a value of 0.931. The value of the MR-QAP regression analysis is 0.592, suggesting that the semantic network of sample 1 had a 59.2% positive influence on that of sample 2. Combined with the two semantic network graphs and degree centrality analysis of two samples, the Chinese public intensively debated the issues of vaccines and vaccination both before and after the marketing approval, so the correlation is high. The debate topics slightly changed after the vaccines were approved for marketing that people cared more about vaccine production than foreign vaccines. In other words, although the issues of vaccines and vaccination are critical public concerns all the time, the launch of approved vaccines stimulated new public concerns on the practical issues of vaccination and the promotion of vaccines.

**Table 2:** Correlation and regression analysis of two semantic networks.

	**QAP correlation**	**MR-QAP regression**
Obs Value	0.931^*^	0.592^**^

* *P = 0.003 < 0.05*

** *P = 0.000 < 0.001*.

### Sentiment Analysis

Based on the sentiment analysis, Table 3 shows the results of the two sentiment classifications of the two samples. We used the percentage value for better comparison between the two samples since sample 2 is more significant than sample 1. Regarding the three sentiment orientations, texts recognized as positive emotions accounted for about 51.75% of sample 1 and 61.61% of sample 2. After the domestic COVID-19 vaccines were officially approved for marketing, the positive emotions of the public increased apparently. The results show that the neutral emotions decreased significantly from 19.36 to 9.28%, and the negative emotions changed from 28.89 to 29.11%. Although the number of Weibo texts with negative emotions in the two samples is similar, there is still a slight increase in the negative emotions after the official approval for marketing.

**Table 3:** Results of sentiment analysis.

	**Sentiment orientation**	**Volume**	**%**	**Emotion**	**Volume**	**%**
Sample 1	Positive	31,113	51.75%	Good	107,517	49.10%
				Happy	23,399	10.68%
	Neutral	11,639	19.36%	Surprise	13,330	6.09%
				Disgust	13,207	6.03%
	Negative	17,370	28.89%	Fear	16,351	7.47%
				Anger	16,342	7.46%
				Sadness	28,845	13.17%
Sample 2	Positive	48,893	61.61%	Good	28,2101	55.24%
				Happy	51,365	10.06%
	Neutral	7,365	9.28%	Surprise	30,369	5.95%
				Disgust	22,987	4.50%
	Negative	23,101	29.11%	Fear	46,655	9.14%
				Anger	26,083	5.11%
				Sadness	51,094	10.00%

Table 3 also shows the result of the seven specific emotions classifications according to the DLUT sentiment dictionary, and we corresponded the particular emotions to the above three sentiment orientations. Concerning that the number of the two samples is not equal, we compared them with the transformed percentages. In the positive emotions, the words reflecting “good” accounted for the highest proportion in both samples, reaching 49.10% in sample 1 and increasing to 55.24% in sample 2. As for the negative emotions, the proportion of the words reflecting “fear” increased from 1.67 to 9.14% after the vaccines were approved for marketing. Unlike the increase of the emotions of “good” and “fear,” the other five emotions all decreased to varying extents. The emotions of “happy” and “surprise” showed a slight decrease, indicating a relatively stable state in the two samples. The emotions of “disgust,” “anger,” and “sadness” decreased significantly, and the sum of their decrease reached 7.05%.

Regarding the sentiment classification analysis above, there existed apparent sentiment changes after the domestic COVID-19 vaccines were approved for marketing. The Chinese public sentiment orientations became more polarized after the marketing approval. Weibo texts with neutral emotions significantly decreased, accounting for <10%, texts with positive emotions increased to 61.61%, and those with negative emotions increased to 29.11%. There was a specific correlation between the sentiment orientation changes and the number of words reflecting particular emotions. There were 218,991 words with emotions existing in 60,122 Weibo texts of sample 1. Each text contains 3.64 emotion words on average; 510,654 emotion words exist in 79,359 Weibo texts of sample 2, which includes 6.43 emotion words per text. The texts from sample 2 contain a higher number of emotional words, indicating that the Chinese public expressed their attitudes and emotions more directly and clearly after the vaccines were approved for marketing. In addition, the neutral emotions shrunk rapidly and separated into positive or negative emotions after the approval.

Many factors affected the changes in sentiment orientations. This study mainly explored the factor of the number of emotion words in the two sample texts. Table 4 further analyzes the exact words with “good” and “fear.” The number of words with “good” emotion reached 282,101, which contributed to the increased positive emotions, and there are many words with “fear” emotion, promoting negative emotions.

**Table 4:** High-frequency words of “good” and “fear” in the two samples.

**NO**.	**Sample 1**				**Sample 2**			
	**Good**	**Frequency**	**Fear**	**Frequency**	**Good**	**Frequency**	**Fear**	**Frequency**
1	Vaccines	1,539	Vaccines	1,491	Vaccines	1,688	Vaccines	1,613
2	COVID-19	1,271	COVID-19	1,284	COVID-19	1,114	COVID-19	1,311
3	Vaccination	577	China	693	Vaccination	941	Vaccination	798
4	Marketing	308	Medicament	527	Free	908	China	603
5	China	281	Safe	518	Large-scale	320	Effect	332
6	Hope	212	Trial	515	Production Capacity	294	Pfizer	302
7	Clinic	183	Clinic	514	Nationwide	169	Side-effect	293
8	Nation	179	Vaccination	496	Thank	169	Domestic	271
9	Success	165	Precedence	332	Government	143	Safe	224
10	Domestic	161	Crowd	324	Satisfy	139	America	218

In addition, we conducted an aggregate analysis of emotions and texts in this paper. As referred above, “good” and “fear” had a more significant impact on the proportion of positive and negative emotions. To further explore the specific text content associated with each emotion, this study counted the number of emotion words in each text and extracted 1,000 posts with the highest expression of “good” and 1,000 posts containing “fear” from each sample as four groups of data with a total of 4,000 posts. As shown in Table 4, we counted the word frequency and listed the words with the highest frequency in each data group. It was found that before the vaccines were approved for marketing, the “good” emotion was mainly related to the public expectation of the upcoming approval of vaccines and the success of the current clinical trials. The domestic COVID-19 vaccines of China achieved good trial results, which gained public affirmation and appreciation, and some of them called for the marketing approval of the vaccines. After the vaccines were approved for marketing, the Chinese public highly appreciated the policy of free vaccination to Chinese citizens and showed faith in the national vaccine production capacity. These related texts are all with “good” emotion. The texts involved with “fear” emotion showed some differences in the two samples. Before the marketing approval, the public concerns concentrated on the safety of vaccines and priority groups for vaccination. Some refused to be the first batch to take the vaccines because they doubted the safety of the vaccines; someone believed in the vaccines but refused to be the priority group with a low risk of COVID-19 infection with those who worked in the frontline. After the marketing approval, texts with “fear” emotion are mostly connected to the side effects of the vaccines, and the public also cites the cases of Pfizer vaccines to express their fear of the side effects.

## Discussion

First, the official announcement on the approval of COVID-19 vaccines for marketing improved the Chinese public acceptance of domestic vaccines. In the semantic network analysis, the public focused more on the safety and effectiveness of the vaccines before the official approval for marketing, and they were more concerned about whether the vaccines are suitable for practical application. After the vaccines were approved for marketing, the focus of the public attention switched to the process of vaccination, the production capacity, and the scale of vaccine production, reflecting that the public was more concerned about the practical issues of vaccination. The shift of the public concerns shows that the official press release of the approved vaccines improved the public recognition and acceptance of domestic COVID-19 vaccines. According to the specific sentiment analysis, the public emotional inclination from their online posts about domestic vaccines has changed after the official announcement. Positive emotions were raised by 9.86%, especially the “good” emotion, which increased significantly by 6.14%. The content of texts with “good” emotion shows that the public appreciated the free vaccination policy of the government and the efforts to expand production capacity. The public sentiment orientation indicates that the official announcement of the approval of the vaccine for marketing and the related policies of vaccination and vaccine production positively promoted public acceptance of the domestic COVID-19 vaccines.

After the state council information office of PRC released that the Sinopharm inactivated vaccine from China has been approved for conditional marketing, national authorized media such as Xinhua News Agency, People's Daily, CCTV news, and other local media reported the news then. This official news and reports mainly contained the following information: (1) reporting that the approved vaccines have experienced a strict process of review, recheck, verification, retest, and data support, enhancing that the decision of conditional marketing is scientific and systematic to ensure the safety of the vaccine; (2) reporting the effectiveness of the approved vaccines that the protective efficacy reaches 79.34%, which meets the applied standard of WHO and the State Food and Drug Administration; (3) reporting the vaccination program that vaccination is free and voluntary, priority groups will be taken in batches under the premise of the informed consent, and the exclusion of any contraindication, with an ultimate goal of herd immunity; (4) reporting that China has the ability of large-scale production of the vaccines to ensure the vaccines production capacity (36). The news introduced the safety and effectiveness of the vaccine, the vaccination program, and the production capacity to the public. Combined with the changes in public emotions and public concerns on Weibo posts, it can be inferred that the official releases positively impacted the change in the public attitudes to domestic COVID-19 vaccines. This conclusion further supports existing research findings that official news releases with complete and targeted information are essential in promoting public acceptance of vaccines (37, 38).

Safety and effectiveness are vital factors influencing public vaccine hesitancy. The WHO identifies the behavior of “delayed or refused vaccination despite available vaccine services” as vaccine hesitancy and believes that the increasing vaccine hesitancy has become a significant threat to global health (39). Some researchers argued that vaccine safety is a crucial factor influencing vaccine hesitancy (5), the side effects, safety, and duration of protection of vaccines influence the vaccination preference of an individual (10, 40).

In the previous investigation on COVID-19 vaccination, the factors that contributed to vaccine hesitancy included doubts about the safety and effectiveness of vaccines (41, 42), which also influence the vaccination willingness of the people (9).

The semantic network in this study showed that the public was most concerned about the safety and effectiveness of vaccines before the official announcement. The debate about the priority groups for vaccination reflects safety concerns to some extent. As seen in the sentiment analysis, although the positive emotions toward the vaccines increased in general after the official announcement for conditional marketing, the “fear” emotion is the only negative emotion that increased instead of decreasing. In terms of semantics, the keywords of the “fear” emotion are mainly about the side effects of the vaccines. The comparison with side effects of foreign COVID-19 vaccines like Pfizer was driven online to discuss whether domestic COVID-19 vaccines would have the same problems. Although the official press releases introduced developing vaccines and the data of tests, some of the public reiterated their doubts about the safety and effectiveness of domestic vaccines.

Lastly, stimulus generalization existed in the Chinese public attitudes to domestic COVID-19 vaccines. Stimulus generalization, derived from Pavlovic's conditional response experiment, refers to the behavioral responses of an organism to specific stimuli with certain conditions attached, which can be evoked again under the same or similar stimuli (43). Stimulus generalization emphasizes that the same or similar stimulation can produce the same or similar behavioral response for an organism. The same or similar comments and reports can cause the same or similar reactions to the audience and the network public opinion.

It should be noted that the positive emotion of the public to the domestic COVID-19 vaccines increased after the official announcement of the conditional marketing, but “fear,” a negative emotion, increased rather than decreased at the same time. In the semantic network analysis, the “fear” after the marketing approval was mainly related to keywords of “side effects,” “adverse reactions,” and “Pfizer,” which is due to the mass news reports about the side effects of foreign vaccines, especially those produced by Pfizer, that caused deaths. For example, CCTV International (44), Tencent News (45), and other media reported that “Portuguese medical worker died 2 days after taking Pfizer-BioNTech COVID-19 vaccine”; Xinhua News (46) reported “Nearly 4,400 adverse events reported in the U.S. after receiving Pfizer-BioNTech COVID-19 vaccine”, and so forth. Besides, the news was reported by the dominant media and other media, recognized with authority in China. Although the reported cases of side effects, even death, were all about the foreign vaccines, it still causes the stimulus generalization among the public regarding any COVID-19 vaccines. In other words, the negative public emotions caused by the foreign vaccines would also be generalized to the domestic vaccines with fear, which also further responds to an existing finding that adverse media reports on vaccines will affect public attitudes to the whole vaccines (47).

## Conclusion

This article obtained the data of Weibo texts that reflected the public attitudes toward the domestic COVID-19 vaccines around 1 week before and after the official announcement of approved vaccines for conditional marketing with the data mining methods. This article employed semantic network and sentiment analysis to track the public attitudes and the changes of their attitudes toward the approved vaccines. Data mining and analysis based on artificial intelligence have advantages in coverage and data processing compared with questionnaires and personal interviews. The large monthly active users of Sina Weibo, which reached 500 million, can reflect the Chinese public attitudes and their changes toward domestic COVID-19 vaccines. The findings in this study can further provide suggestions for Chinese governments, public health administrations, and media to promote vaccine information and the public willingness to vaccination.

## Limitations

Although this paper employs convenient research methods and tools based on big data, this study has some limitations. On one hand, the research samples selected in this study are not fully representative of the general public. They only represent the opinions of those who are willing to express themselves online. On the other hand, our research is limited to a description of the public attitudes expressed by Weibo users. However, the relation between the public attitudes to vaccines and the actual vaccination behavior deserves further study, such as combining big data with traditional research methods. For the application of big data in research, there is a need to further expand the data sources and data mining capabilities, especially for research on the correlation between vaccine attitudes and influencing factors.

## Data Availability Statement

The raw data supporting the conclusions of this article will be made available by the authors, without undue reservation.

## Ethics Statement

This study was reviewed and approved by the Institutional Boards at the two participating institutions: Nanjing Normal University and Shanghai Normal University. Written informed consent for this study was not required in accordance with local legislation and national guidelines.

## Author Contributions

All authors listed have made a substantial, direct and intellectual contribution to the work, and approved it for publication.

## Funding

This work was supported by grants from the National Social Science Foundation of China (17CXW016).

## Conflict of Interest

The authors declare that the research was conducted in the absence of any commercial or financial relationships that could be construed as a potential conflict of interest.

## Publisher's Note

All claims expressed in this article are solely those of the authors and do not necessarily represent those of their affiliated organizations, or those of the publisher, the editors and the reviewers. Any product that may be evaluated in this article, or claim that may be made by its manufacturer, is not guaranteed or endorsed by the publisher.
